# Optimization of Bleaching Process and Evaluation of Pulp Performance for Super-Arundo Donax Kraft Pulp

**DOI:** 10.3390/polym18060750

**Published:** 2026-03-19

**Authors:** Zhangming Cai, Xingxiang Ji, Jie Liang, Zhongjian Tian, Jingpeng Zhou

**Affiliations:** 1 State Key Laboratory of Green Papermaking and Resource Recycling, Qilu University of Technology (Shandong Academy of Sciences), Jinan 250353, China; 2 Shandong Huatai Paper Co., Ltd., Dongying 257335, China

**Keywords:** Super-Arundo donax, oxygen delignification, bleaching, paper properties, model construction

## Abstract

With the increasing emphasis and protection on forest resources worldwide, the development of non-wood plant fiber raw materials has become a key path to promote the green and sustainable development of China’s pulp and paper industry. In this study, Super-Arundo donax, a new non-wood fiber raw material, was systematically investigated for its applicability in the bleaching process. Firstly, by adjusting key bleaching technical variables such as alkali dosage, time, oxygen pressure and temperature, the oxygen delignification process of the Super-Arundo donax kraft pulp was optimized. The data revealed that under the experimental conditions of 3.0% alkali dosage, 60 min bleaching time, 100 °C bleaching temperature, 0.6 MPa oxygen pressure and 0.6% MgSO_4_ dosage, the bleached pulp yield reached 91.58%, the brightness was 42.04% ISO, and its tensile index was 60.92 N·m/g, bursting index was 4.16 kPa·m^2^/g, and tear index was 5.45 mN·m^2^/g, respectively. To further enhance the bleaching effect, the study introduced the H_2_O_2_ enhanced oxygen delignification process. The alkali dosage, bleaching temperature and H_2_O_2_ dosage were selected as the process parameters, with the pulp yield and brightness as the response indicators. A central composite design was adopted to construct a response surface model, and the interaction effects among various factors were analyzed. The optimized optimal process conditions are as follows: pulp concentration 10%, alkali dosage 2.84%, bleaching temperature 105 °C, H_2_O_2_ dosage 4.85%, bleaching time 60 min, MgSO_4_ dosage 0.6%. Under these conditions, the pulp yield was 89.76% and the brightness reached 53.85% ISO. Therefore, Super-Arundo donax possesses excellent pulp-making and papermaking properties, and is expected to serve as a high-quality non-wood fiber raw material to alleviate the pressure on traditional papermaking raw materials and contribute to the green, sustainable and low-carbon transformation of the pulp and paper industry.

## 1. Introduction

The papermaking industry, a pillar raw material industry of the national economy, is undergoing a crucial transformation in its raw material supply structure from “traditional dependence” to “diversified expansion”. For a long time, wood (softwood and hardwood) has served as traditional papermaking raw materials. Wood possesses stable fiber morphology and regular lignin structure. These advantages promote the mature development of global pulp bleaching technology. A typical process system is thus established to balance delignification efficiency and fiber protection. However, with the tightening of global forest resource protection policies, wood resources have become limited and costs have risen (the import volume was approximately 30 million tons in 2023) [1,2], and it is urgent to expand the sources of papermaking raw materials to non-wood fibers [3,4].

Currently, materials such as oil palm empty fruit bunches [5], sugarcane [6], wheat straw [7], and Pennisetum purpureum [8] have been explored for their papermaking potential. Super-Arundo donax is a promising non-wood raw material for pulping and papermaking. It can adapt to cold and saline-alkali environments and shows strong disease and pest resistance. Consequently, it requires lower fertilizer input and incurs reduced production costs throughout its entire growth cycle compared with other gramineous plants, making it a cash crop with excellent economic benefits. Meanwhile, the Super-Arundo donax contains over 40% cellulose and 25% hemicellulose, rendering it an ideal material for the pulp and paper industry [9,10,11,12]. Existing research on Super-Arundo donax has explored its application in producing high-strength paper via semi-chemical pulping. Under optimized conditions (with the mass ratio of NaOH to Na_2_CO_3_ set at 4:1, the volume fraction of N_2_O controlled at 12%, the cooking temperature maintained at 130 °C, the cooking time fixed at 90 min, and solid-to-liquid ratio adjusted to 1:6), the prepared paper showed outstanding mechanical properties. The tensile index, tear index, and ring crush index reached 30.5 N·m/g, 6.08 mN·m^2^/g, and 11.6 N·m/g [13]. Additionally, binder-free fiberboards fabricated via steam explosion have been successfully applied in the construction materials industry. At a pretreatment temperature of 200 the resultant fiberboards achieved average values of 4514 MPa for modulus of elasticity (MOE), 34.51 MPa for modulus of rupture (MOR), 4.125 MPa for internal bonding strength (IB), 8.68% for 24 h thickness swelling (TS), and 9.22% for 24 h water absorption (WA). These performance metrics were comparable to those of commercially available fiberboards [14]. However, the research reports on kraft pulping followed by bleaching of this raw material remain limited.

Kraft pulping uses NaOH and Na_2_S as the main components of cooking liquor. The presence of sulfide ions (S^2−^) and hydrosulfide ions (HS^−^) inhibits the secondary condensation of lignin structural units. Notably, the phenolic lignin in gramineous raw materials has an unstable structure, with H-type lignin being the most susceptible to degradation. Therefore, compared with other pulping methods, kraft pulping is more conducive to the removal of lignin molecules. Notably, oxygen delignification is a key stage in the pulping and papermaking bleaching technologies. Oxygen acts as an oxidant to react with phenolic lignin, generating superoxide anion radicals (·O_2_^−^), hydroperoxyl radicals (HOO·), and hydroperoxide anions (HOO^−^) [15], which will destroy the aromatic ring structure of residual lignin in the pulp and convert it into soluble small molecules. Therefore, it has the characteristics of chlorine-free pollution, efficient removal of lignin, and minimal fiber damage [16]. Using oxygen delignification, Tavares et al. bleached eucalypt kraft pulp and achieved a reduction in Kappa number from 12.3 to 10.6 and the intrinsic viscosity from 1185 cm^3^/g to 1140 cm^3^/g [17]. Zhao et al. integrates high-dose ClO_2_ coupled with oxygen, and their results showed that: the DPI-O process (with ClO_2_ applied first followed by oxygen treatment) could oxidize lignin into quinonoid intermediates and then further degrade them, achieving a high delignification rate of 84.90%, reducing the Kappa number and AOX generation, and the resultant pulp attained a brightness of 49.98% [18]. This not only achieves efficient lignin removal and initial improvement in pulp whiteness, but also avoids the generation of chlorine-containing pollutants at the source. Notably, the use of H_2_O_2_ to enhance the oxygen delignification process (OP) can significantly improve the pulp’s delignification effectiveness and brightness, while selectively oxidizing the lignin [19,20,21,22]. Li et al. observed that following alkaline hydrogen peroxide (AHP) pretreatment, the residual lignin was characterized by S-unit and β-O-4 linkage enrichment, accompanied by relative reductions in ferulic acid (FA) and p-coumaric acid (PCA) structures, an increase in carboxylic hydroxyl groups, and a decline in phenolic hydroxyl content [23]. Li et al. applied hydrogen peroxide bleaching to the CTMP of poplar, increasing its brightness from 46.3% to 77.5% [24]. However, based on the current literature, limited studies have been conducted on the bleaching of Super-Arundo donax so far. Oxygen delignification-based bleaching can enrich and expand the application scope of non-wood raw materials in the pulp and paper field.

This study analyzes and investigates the bleaching process parameters of Super-Arundo donax kraft pulp. Firstly, the impacts of alkali charge, bleaching temperature, bleaching time, and oxygen pressure on the oxygen delignification of Super-Arundo donax kraft pulp were explored by changing one variable at a time. Comprehensive analysis of various pulp properties was performed to determine the optimal process conditions. Finally, three influencing factors-alkali charge, temperature, and H_2_O_2_ dosage were used to analyze the effect of H_2_O_2_-enhanced oxygen delignification on Super-Arundo donax kraft pulp, and a model was established using the response surface methodology (RSM) with Design-Expert software (version 13). This work provides new insights for the development of the application of non-wood raw material Super-Arundo donax in the bleaching stage and demonstrates its potential in the field.

## 2. Materials and Methods

### 2.1. Materials

Century Sunshine Paper Co., Ltd. (Weifang, China) provided Super-Arundo donax. It was air-dried at room temperature, crushed into bamboo chips with a length of 3~5 cm, washed, air-dried again, and then stored in sealed bags for subsequent experiments.

All chemicals used were of analytical purity. Reagents used in the experiment: Sodium hydroxide (NaOH), sulfuric acid, anhydrous magnesium sulfate (MgSO_4_), hydrogen peroxide (H_2_O_2_), potassium iodide (KI), sodium sulfide (Na_2_S), Cupriethylenediamine, sodium thiosulfate (Na_2_S_2_O_3_), and potassium permanganate (KMnO_4_) were all bought from Shanghai Maclin Biochemical Co., Ltd. (Shanghai, China). Shandong Zhumeng Economic and Trade Development Co., Ltd. (Jinan, China) provided oxygen.

### 2.2. Super-Arundo Donax Kraft Pulp

Super-Arundo donax was placed in a vertical rotating autoclave (No. 2615, KYORAKU CO., LTD., Tokyo, Japan), and the cooking process was carried out under adjusted operating parameters. Intense reaction conditions facilitated lignin dissolution but concurrently induced excessive cellulose degradation. In contrast, mild reaction conditions resulted in insufficient pulping efficiency and compromised overall process performance. Consequently, the optimal conditions were determined as follows: solid-to-liquid ratio of 1:4, sulfidity of 25% (calculated as NaOH), alkali dosage of 22% (calculated as NaOH), 1.5 h cooking duration, and 165 °C cooking temperature. After cooking, the pulp was collected and washed in a 300-mesh bag until the filtrate became neutral. In this way, Super-Arundo donax kraft pulp was obtained. The pulp was then sealed in plastic bags for moisture equilibration. Subsequent bleaching experiments were performed using the prepared Super-Arundo donax kraft pulp as the raw material.

### 2.3. Oxygen Delignification Bleaching of Super-Arundo Donax Kraft Pulp

The bleaching process of kraft pulp was investigated in a horizontal rotating digester (KRK, Model No. 11, Japan), with the aim of optimizing its technological parameters. For each bleaching experiment, 100 g of oven-dried pulp was used, and deionized water was added to adjust the pulp consistency to 10%, with MgSO_4_ dosage fixed at 0.6%. Bleaching was conducted under varying conditions, including alkali dosage (2, 2.5, 3, 3.5, and 4%), temperature (80, 90, 100, 110, and 120 °C), duration (20, 40, 60, 80, and 100 min), and oxygen pressure (0.4, 0.5, 0.6, 0.7, and 0.8 MPa). After adding the pulp and required chemicals, the digester was connected to an oxygen cylinder for gas injection. Once the preset oxygen pressure was reached, the gas was vented after maintaining the pressure for 1 min, and this venting procedure was repeated three times. Upon completion of bleaching, the pulp–water mixture was transferred to a 300-mesh pulp bag. The bleached pulp was thoroughly washed yielding Super-Arundo donax kraft bleached pulp. After dispersion, the pulp was placed for 24 h.

### 2.4. H_2_O_2_-Enhanced Oxygen Delignification Bleaching

#### 2.4.1. Model Design for H_2_O_2_-Enhanced Oxygen Delignification Bleaching

A total of 17 sets of experiments for H_2_O_2_-enhanced oxygen delignification was designed using Design-Expert software (Supplementary Table S1). Alkali dosage, temperature, and H_2_O_2_ dosage were selected as the influencing factors in this study. The experiments were conducted in a horizontal rotating digester (KRK, Model No. 2611, Japan), with 60 g of oven-dried pulp used per experiment. The fixed process parameters were set as follows: oxygen pressure of 0.6 MPa, pulp consistency of 10%, MgSO_4_ dosage of 0.6%, and bleaching duration of 60 min. In accordance with the conditions specified by the software, sodium hydroxide and H_2_O_2_ were added, and the bleaching temperature of the digester was set accordingly. Upon completion of the experiments, the pulp was collected and thoroughly washed. After dispersion, the pulp was placed in a sealed bag for moisture equilibration.

#### 2.4.2. Model Equations

Using alkali dosage, temperature, and H_2_O_2_ dosage as independent variables, empirical models for pulp yield (PY) and brightness (BR) were established. The following equations can be used to express this relationship:

(1) $$y\;={\;\varphi }_{0}\;+\;\varphi_{1}x_{1\;}+\;\varphi_{2}x_{2}\;+\;\varphi_{3}x_{3}\;+{\;\varphi }_{4}x_{1\;}^{2}+\;\varphi_{5}x_{2}^{2}\;+\;\varphi_{6}x_{3}^{2}\;+\;\varphi_{7}x_{1}x_{2}\;+\;\varphi_{8}x_{1}x_{3}\;+{\;\varphi }_{9}x_{2}x_{3}$$

This is a multiple nonlinear regression model designed to describe the complex nonlinear relationships among the three independent variables. In this experiment, x_1_, x_2_ and x_3_ represent alkali dosage (*A*), temperature (*T*), and H_2_O_2_ dosage (*H*), respectively. Thus, the empirical models for pulp yield (PY) and brightness (BR) can be expressed as follows:

(2) $$y\;={\;\varphi }_{0}\;+\;\varphi_{1}A\;+\;\varphi_{2}T\;+{\;\varphi }_{3}H\;+\;\varphi_{4}A^{2}\;+\;\varphi_{5}T^{2\;}+\;\varphi_{6}H^{2}\;+\;\varphi_{7}AT\;+{\;\varphi }_{8}AH\;+\;\varphi_{9}TH$$

#### 2.4.3. Model Analysis and Validation

The reliability and fitness of the established models were evaluated via analysis of variance (ANOVA), while the significance of individual terms within the models was determined using *p*-values. The coefficient of determination (*R*^2^), adjusted coefficient of determination ($R_{adj}^{2}$) and predicted coefficient of determination ($R_{pre}^{2}$) were employed to comprehensively assess the model’s fitting performance.

### 2.5. Pulp Characterization and Analysis

The acid-soluble lignin content was measured following GB/T 10337-2008. The acid-insoluble lignin content was tested in accordance with GB/T 747-2003. The intrinsic viscosity in cupriethylenediamine (CED) solution was determined based on GB/T 1548-2016 [6]. The monosaccharide contents of bleached pulp were determined according to the NREL/TP-510-42618 protocol l [13].

### 2.6. Characterization Methods for Physical Properties of Paper

Deionized water was added to 30 g of oven-dried bleached pulp to bring the pulp consistency to 10%. To achieve a beating degree of 40 ± 2 °SR, the pulp was beaten in a PFI mill (RISE PFI AS, Trondheim, Norway). An appropriate amount of pulp was placed in a fiber disintegrator (Lorentzen & Wettre, Kista, Sweden) and disintegrated at 3000 r/min for 2 min. to form a uniform pulp suspension. We made 100 g/m^2^ circular handsheets with a (Frank-PTI GmbH, Frankfurt am Main, Germany).

In accordance with the corresponding Chinese national standards (GB/T), the physical properties of the handsheets were determined, using specialized testing instruments as follows:

Tensile strength: measured with a horizontal tensile tester (Model XLW-B, Lorentzen & Wettre, Kista, Sweden) in compliance with GB/T 12914–2018. Tear strength: determined using a paper tear tester (Model SLY-1000, Lorentzen & Wettre, Kista, Sweden) following GB/T 455–2002. Brightness: measured using a paper brightness tester (Lorentzen & Wettre, Kista, Sweden) in accordance with GB/T 7974–2013 [2].

### 2.7. Characterization

At an accelerating voltage of 10 kV, the surface morphologies of different Super-Arundo donax paper sheets were observed using a scanning electron microscope (SEM, TM4000Plus, Hitachi High-Tech Corporation, Tokyo, Japan) [25]. Prior to SEM observation, the dried samples were sputter-coated with a thin layer of gold (≈10 nm) using an ion sputter coater (e.g., Quorum Technologies Co., Ltd. Q150R ES, Laughton, East Sussex, UK) to enhance electrical conductivity. The effects of chemical reagents on fibers were evaluated by comparing morphological features across samples:

Fiber integrity: assessing whether excessive alkali or high temperature caused fiber fragmentation, kinking, or fibrillation (e.g., presence of broken fiber ends or collapsed cell walls).

Microstructural changes: identifying evidence of hemicellulose/lignin dissolution (e.g., exposed fibrils) or cellulose degradation (e.g., surface cracks) under high magnification, which reflects the balance between delignification efficiency and fiber damage.

FTIR spectra were recorded on a Bruker FTIR spectrometer (INVENIO, Bruker Corporation, Ettlingen, Germany) across the wavenumber range of 4000~500 cm^−1^ to analyze the variations in functional groups of unbleached pulp, oxygen-delignified pulp, and H_2_O_2_-aided oxygen-delignified pulp [26].

The X-ray diffraction (XRD) patterns of pulps treated under different conditions were determined using an X-ray diffractometer. The measurement was performed with Cu-Kα radiation as the target, operating at a voltage of 40 kV, a current of 40 mA, a scanning speed of 2.0°/min, and a scanning range of 5~60° [27].

## 3. Results

### 3.1. Oxygen Delignification Bleaching Process of Super-Arundo Donax

The kraft pulp properties were determined as: yield of 45.22%, Kappa number of 22.78, brightness of 21.06% ISO, viscosity of 1282 mL/g, tear index of 1.89 mN·m^2^/g, tensile index of 72.35 N·m/g, and burst index of 4.06 kPa·m^2^/g.

#### 3.1.1. Effect of Alkali Charge on Oxygen Delignification of Super-Arundo Donax Kraft Pulp

To investigate the effect of alkali charge on the oxygen delignification of Super-Arundo donax kraft pulp, other process conditions were fixed as follows: time of 60 min, temperature of 100 °C, magnesium sulfate dosage of 0.6%, and oxygen pressure of 0.6 MPa. Only the alkali charge was varied to obtain experimental data, and the results were analyzed. The specific experimental results are presented in Supplementary Table S2.

The essence of oxygen delignification lies in oxygen-alkali bleaching, whereby alkali dosage influences the efficiency of both the overall delignification stage and carbohydrate degradation. Alkali functions to activate lignin by reacting with phenolic hydroxyl groups and enol groups within lignin, forming more reactive phenolates and enolates that subsequently undergo reaction with oxygen [28]. Under alkaline conditions, oxygen reacts with ring-conjugated carbonyl groups in the lignin structure to form dioxetane moieties. Ultimately, the rearrangement of these cyclic dioxane peroxides results in the cleavage of C_α_ and C_β_ linkages.

**Figure 1:**
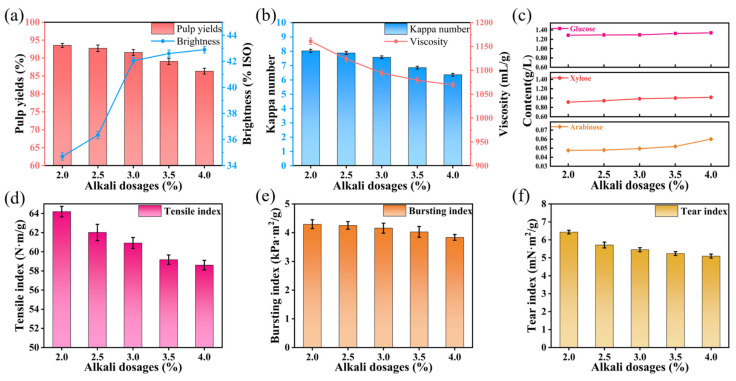
Effects of different alkali dosages on oxygen delignification of Super-Arundo donax kraft pulp. (**a**) Pulp yield and whiteness; (**b**) Kappa number and viscosity; (**c**) contents of arabinose, xylose, and glucose in pulp; (**d**) tensile index; (**e**) bursting index; (**f**) tear index.

When the alkali dosage is 2.0% in Supplementary Table S2, the yield of bleached pulp reaches 93.55%, viscosity reaches 1161 mL/g, the brightness is 34.7% ISO, and Kappa number is 8.03. It is noteworthy that, as shown in Figure 1a, when the alkali dosage increases from 2.0% to 3.0%, the yield decreases gradually, but the brightness rises rapidly to 42.04% ISO. However, when the alkali dosage increases from 3.0% to 4.0%, the brightness only increases by 0.85% ISO, while the yield decreases more rapidly to 86.34%. A higher alkali dosage increases the concentration of the bleaching solution. The stronger concentration gradient promotes the diffusion and penetration of the liquor, thus accelerating the reaction with pulp. A more intense reaction leads to more dissolution of organic substances, resulting in a lower pulp yield. The Kappa number, which reflects the residual lignin content in pulp, also acts as an indirect measure for characterizing the extent of delignification; a lower pulp Kappa number means lower lignin content. In contrast, viscosity can characterize the degree of cellulose degradation during the bleaching process, where a lower viscosity indicates a more significant degree of cellulose degradation. As shown in Figure 1b, the rate of viscosity decline follows a concave downward curve, meaning that while delignification occurs, the plant fibers are also being damaged. When the alkali dosage is 3.0%, the pulp has a viscosity of 1094.5 mL/g and a Kappa number of 7.58. Increasing the alkali dosage to 3.5% reduces the viscosity by 1.37%, and further increasing the dosage to 4.0% decreases the viscosity by 0.93% to 1069.5 mL/g. The contents of arabinose, glucose, xylose, and lignin in the pulp were also analyzed. The glucose content roughly reflects the cellulose content of the pulp, which, as shown in Figure 1c, increases with the alkali dosage. The arabinose content is minimal, reaching only 0.0600 g/L even when the dosage increases to 4.0%. Lignin, divided into acid-soluble and acid-insoluble fractions, decreases as the alkali dosage increases, indicating that the oxygen delignification is effective [29].

Subsequently, the effect of different alkali dosages on the strength of paper made from bleached pulp was discussed. The physical strength of paper not only depends on the strength of individual fibers, but is also impacted by inter-fiber bonding strength [30]. From Figure 1d–f, the tensile index, burst index, and tear index of the paper all show a decreasing trend because the fibers are partially damaged during the lignin removal process, resulting in a decrease in the intrinsic strength of the fibers. When the alkali dosage is 2.0%, the paper has a burst index of 4.30 kPa·m^2^/g, a tensile index of 64.19 N·m/g, and a tear index of 6.43 mN·m^2^/g. Increasing the alkali dosage to 3.0% results in a slight decrease in the physical properties of the paper, with a tensile index of 60.92 N·m/g, a burst index of 4.19 kPa·m^2^/g, and a tear index of 5.45 mN·m^2^/g. A lower pulp yield can affect the economic efficiency for manufacturers. Considering factors such as pulp brightness and the paper’s physical performance, an alkali dosage of 3.0% was selected for the oxygen bleaching of sulfate pulp from Super-Arundo donax kraft pulp.

#### 3.1.2. Effect of Temperature on Oxygen Delignification of Super-Arundo Donax Kraft Pulp

To investigate the effect of temperature on the oxygen delignification of Super-Arundo donax kraft pulp, other process conditions were fixed as follows: time of 60 min, alkali charge of 3%, magnesium sulfate dosage of 0.6%, and oxygen pressure of 0.6 MPa. Only the temperature was varied to obtain experimental data, and the results were analyzed. The specific experimental results are presented in Supplementary Table S3.

**Figure 2:**
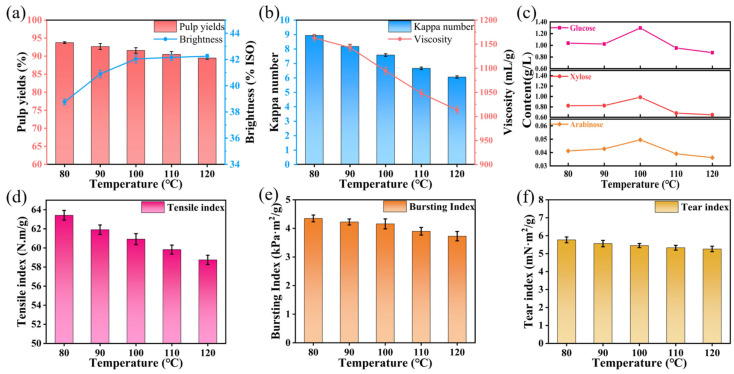
Effects of different temperatures on oxygen delignification of Super-Arundo donax kraft pulp. (**a**) Pulp yield and whiteness; (**b**) Kappa number and viscosity; (**c**) contents of arabinose, xylose, and glucose in pulp; (**d**) tensile index; (**e**) bursting index; (**f**) tear index.

Under the conditions of fixed alkali charge and time, the yield of bleached pulp reached 93.76% at a temperature of 80 °C. With the increase in temperature, the yield began to decrease, and when the temperature reached 120 °C, the yield was only 89.48%. It can be observed from Figure 2a that the whiteness had already reached 42.04% ISO at 100 °C, and there was almost no change in whiteness with further temperature increase. In the initial stage of bleaching, the activation energies of lignin and carbohydrates are 10 kJ/mol and 40 kJ/mol, respectively, while in the subsequent bleaching stage, the activation energies of lignin and carbohydrates are 45 kJ/mol and 53 kJ/mol [31]. Therefore, when the temperature is too high, the selectivity of the lignin removal reaction is greatly reduced. When the temperature exceeds 120 °C, the cellulose and hemicellulose in the pulp will be over-oxidized, followed by peeling reactions, thus lowering the viscosity of carbohydrates significantly [32]. The downward trend of viscosity in Figure 2b exactly reflects this situation.

The effect of temperature on oxygen delignification and bleaching process is mainly reflected in changing the molecular motion rate and chemical reaction selectivity. Oxygen delignification involves both electrophilic and nucleophilic reactions, as well as ionic and free radical reactions. Increasing temperature can enhance molecular kinetic energy, raise the collision frequency among oxygen molecules, OH^−^ and lignin macromolecules, and promote the occurrence of free radical reactions. Free radical reactions can fragment lignin macromolecules and degrade them into soluble small molecules, thereby rapidly reducing the kappa number of pulp. Under mild conditions (temperature below 100 °C), elevated temperature mainly intensifies the thermal motion of molecules, accelerating the degradation and dissolution of a large number of reactive lignin structures and, thus, significantly improving pulp brightness. As the temperature further rises to severe reaction conditions, most oxidizable lignin has been removed, the difference in activation energy between lignin and carbohydrates decreases, the selectivity of delignification declines sharply, and the increase in brightness slows down significantly. At this point, hydroxyl radicals and perhydroxyl radicals generated from H_2_O_2_ decomposition also attack carbohydrates, resulting in glycosidic bond cleavage, damage to fiber surface structure and changes in light scattering properties, which further restrict brightness improvement. Meanwhile, high temperature reduces the solubility of oxygen in the liquid phase, accelerates the ineffective decomposition of hydrogen peroxide, and weakens the chelating stabilization of MgSO_4_. Consequently, the increase in brightness is weakened, while carbohydrates are severely degraded and pulp viscosity decreases continuously. The viscosity was 8.94 mL/g when the bleaching temperature was only 80 °C. When the temperature increased from 80 °C to 100 °C, the viscosity decreased by 15.21% to 7.58 mL/g. With a further temperature increase to 120 °C, the viscosity decreased from 7.58 to 6.06 mL/g, with a decrease rate of 20.05%, indicating that temperature has a relatively significant impact on pulp fibers. From Figure 2c, the contents of arabinose, glucose and xylose in the pulp exhibited a trend that first rose and then fell. When the temperature increased from 80 °C to 100 °C, the glucose content increased from 1.0372 g/L to 1.2962 g/L. With a continuous increase in temperature, the glucose content decreased to 0.8752 g/L. Heating softens and loosens the cell wall structure, making polysaccharides more prone to dissolution and hydrolysis, thereby increasing the glucose content in the solution. However, when the temperature exceeds a certain threshold, glucose undergoes thermal degradation and side reactions. For example, glucose undergoes the Maillard reaction with proteins or amino acids in Super-Arundo donax, resulting in a decrease in the final detected glucose content.

As shown in Figure 2d–f, after oxidative delignification, the tensile index of the pulp decreases significantly, especially at higher temperatures where the pulp experiences greater strength loss. At 120 °C, compared with the pulp at 80 °C, the tensile index decreased by 7.35%, from 63.42 N·m/g to 58.76 N·m/g; the burst index dropped from 4.35 kPa·m^2^/g to 3.73 kPa·m^2^/g; and the tear index decreased from 5.77 mN·m^2^/g to 5.26 mN·m^2^/g. Under higher alkali and temperature conditions, tensile strength decreases more sharply than tear strength mainly due to the different contributions of inter-fiber bonding and fiber structural integrity to these two properties. Tensile strength is highly dependent on inter-fiber bonding strength and the number of effective bonding points. Elevated alkali and temperature promote significant delignification, hemicellulose removal, and fiber swelling, which weaken the inter-fiber adhesion and reduce the bonding ability between fibers. Meanwhile, the degradation of carbohydrates further impairs the bonding interface. As tensile strength is extremely sensitive to the bonding state, it declines rapidly. In contrast, tear strength is more determined by fiber integrity, fiber length, and intrinsic fiber strength. Although high alkali/temperature also cause fiber damage, the reduction in fiber integrity and length is relatively mild compared with the rapid loss of inter-fiber bonding. Fibers can still maintain a certain level of structural integrity and frictional resistance during tearing. Therefore, tensile strength, which relies on inter-fiber bonding, decreases more sharply, while tear strength, governed by fiber integrity, decreases more slowly. The increase in temperature causes significant carbohydrate degradation and reduces the hydroxyl groups on the fiber surfaces, leading to a direct and rapid decrease in tensile strength. When the temperature is 100 °C, the pulp’s burst index, tensile index, and tear index are 60.92 N·m/g, 4.16 kPa·m^2^/g, and 5.45 mN·m^2^/g, respectively. Therefore, to achieve good selectivity of the pulp while ensuring the strength properties, it is appropriate to select a temperature of 100 °C for the oxygen delignification of Super-Arundo donax kraft pulp.

#### 3.1.3. Effect of Time on Oxygen Delignification of Super-Arundo Donax Kraft Pulp

To investigate the effect of time on the oxygen delignification of Super-Arundo donax kraft pulp, other process conditions were fixed as follows: alkali charge of 3%, temperature of 100 °C, oxygen pressure of 0.6 MPa, and magnesium sulfate dosage of 0.6%. Only the reaction time was varied to obtain experimental data, and the results were analyzed. The specific experimental results are presented in Supplementary Table S4.

**Figure 3:**
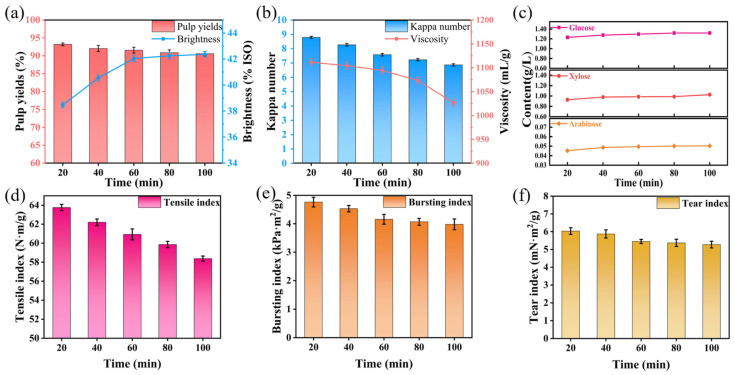
Effects of different time on oxygen delignification of Super-Arundo donax kraft pulp. (**a**) Pulp yield and whiteness; (**b**) Kappa number and viscosity; (**c**) contents of arabinose, xylose, and glucose in pulp. (**d**) tensile index; (**e**) bursting index; (**f**) tear index.

The essence of time’s effect on oxygen delignification lies in regulating the progress of the entire oxygen delignification reaction, thereby balancing the degree of carbohydrate degradation and lignin removal efficiency. Lignin removal proceeds in two stages: a fast stage and a slow stage. This two-stage behavior is caused by the structural differences of lignin. Lignin in pulp can be divided into phenolic and non-phenolic types. Phenolic lignin is highly reactive, while non-phenolic lignin is stable and less reactive. In the initial reaction stage (0~60 min), oxygen and OH^−^ (derived from NaOH) preferentially react with the highly reactive phenolic lignin to rapidly generate lignin free radicals. These free radicals continuously combine with oxygen, gradually cleaving lignin macromolecular chains to form soluble small-molecular fragments that dissolve into the alkaline solution [33]. During this stage, the lignin removal rate (reflected by the reduction range of Kappa number) increases significantly with prolonged time, making it the dominant delignification stage. From 0 to 60 min, pulp yield decreases from 93.20% to 90.58%, and Kappa number drops from 8.79 to 7.58 (Figure 3a,b). From 60 to 80 min, yield decreases by only 0.7% and Kappa number by 0.35 units. This proves the obvious difference in reaction rate between the two stages. When the bleaching duration exceeds 60 min, most of the reactive phenolic lignin has been removed, with the remaining lignin predominantly consisting of recalcitrant non-phenolic lignin characterized by low chemical reactivity. Consequently, the reaction rate between non-phenolic lignin and oxygen as well as OH^−^ is significantly reduced. Even with prolonged reaction time, although the Kappa number continued to decrease to 6.87 at 120 min, carbohydrates underwent excessive degradation, resulting in a substantial decline in pulp viscosity from 1094.5 mL/g (60 min) to 1027 mL/g (120 min) (a reduction of 6.25%). Additionally, some dissolved small-molecular lignin fragments may lose stability and re-adsorb onto the fiber surface. Meanwhile, the concentration of the cooking liquor gradually decreases with the extension of reaction time. As a result, the improvement in whiteness becomes negligible despite prolonged processing—specifically, the whiteness only increased from 42.04% ISO (60 min) to 42.36% ISO (120 min), a mere increase of 0.32% ISO [34]. As illustrated in Figure 3c, arabinose, glucose, and xylose contents rise rapidly in the early stage. They increase from 0.0452, 1.2304, 0.9281 g/L (20 min) to 0.0495, 1.2962, 0.9857 g/L (60 min), respectively. This indirectly confirms that delignification is faster in the early stage of bleaching.

Notably, the severity of carbohydrate degradation is positively correlated with the pulp viscosity reduction, which ultimately leads to the deterioration of paper physical properties—particularly tensile strength. As illustrated in Figure 3d–f, the tensile index of the paper decreased from 63.76 N·m/g (20 min) to 60.92 N·m/g (60 min) (a reduction of 2.84 N·m/g). With a further extension of reaction time to 120 min, pulp fibers were severely damaged, resulting in a tensile index of only 58.37 N·m/g (a total reduction of 5.39 N·m/g compared with 20 min). In contrast, the bursting index decreased moderately from 4.77 kPa·m^2^/g (20 min) to 4.16 kPa·m^2^/g (60 min) and further to 3.98 kPa·m^2^/g (120 min), while the tear index declined from 6.03 mN·m^2^/g (20 min) to 5.45 mN·m^2^/g (60 min) and 5.27 mN·m^2^/g (120 min). Their declining trends were far less pronounced than that of tensile strength. Considering the negligible improvement in pulp whiteness beyond 60 min, combined with the excessive degradation of carbohydrates and the deterioration of paper physical properties caused by prolonged reaction time, 60 min is the optimal oxygen delignification reaction time.

#### 3.1.4. Effect of Oxygen Pressure on Oxygen Delignification of Super-Arundo Donax Kraft Pulp

To investigate the effect of oxygen pressure on the oxygen delignification of Super-Arundo donax kraft pulp, other process conditions were fixed as follows: alkali charge of 3%, temperature of 100 °C, reaction time of 60 min, and magnesium sulfate dosage of 0.6%. Only the oxygen pressure was varied to obtain experimental data, and the results were analyzed. Supplementary Table S5 lists the specific experimental results.

Compared with the other three factors mentioned above, oxygen pressure had a relatively minor impact on the oxygen delignification process. The level of oxygen pressure is related to the interaction between the solid, liquid, and gas phases in the reaction system. Pressure penetration is one of the driving forces for liquor impregnation, and the higher the pressure, the better the liquor impregnation effect. Meanwhile, higher oxygen pressure means more oxygen molecules, which can participate in the reaction more densely and generate more oxygen-derived groups such as superoxide radicals (O_2_^−^·), thereby accelerating lignin removal efficiency. Under conditions of low oxygen pressure and alkali charge, both the degree of lignin removal and carbohydrate degradation are reduced, but the selectivity is high.

**Figure 4:**
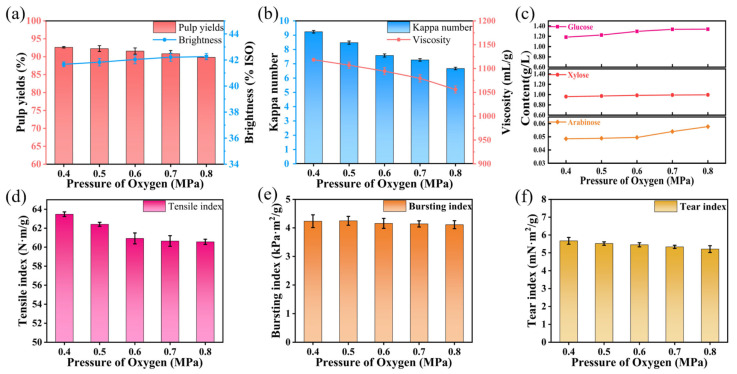
Effects of different pressure of oxygen on oxygen delignification of Super-Arundo donax kraft pulp. (**a**) Pulp yield and whiteness; (**b**) Kappa number and viscosity; (**c**) contents of arabinose, xylose, and glucose in pulp; (**d**) tensile index; (**e**) bursting index; (**f**) tear index.

As shown in Figure 4a–c, oxygen pressure had little effect on pulp yield and brightness. Even when the oxygen pressure reached 0.8 MPa, the yield decreased by 3.04% to 89.82% compared with 0.4 MPa, and the brightness increased by less than 2% ISO. The Kappa number and viscosity decreased slowly, and the contents of various sugars in the pulp showed an upward trend but not significantly, indicating that oxygen pressure had little impact on the amount of lignin removed.

From Figure 4d–f, it is clear that only the tensile index changed significantly before 0.6 MPa, decreasing from 63.47 N·m/g to 60.92 N·m/g, and basically showed no change when the oxygen pressure exceeded 0.6 MPa. The bursting index and tear index hardly changed, fluctuating around 4.16 kPa·m^2^/g and 5.45 mN·m^2^/g respectively. This also indicates that bleaching liquor can penetrate more fully into the gaps of pulp fibers owing to the increase in oxygen pressure, thereby promoting lignin removal, changing the fiber structure, and impairing the inherent strength of the fibers.

Higher oxygen pressure may be combined with shortened reaction time to achieve the same pulp bleaching effect as low oxygen pressure and longer reaction time. However, high pressure will increase the pressure resistance risk of equipment and production energy consumption. Therefore, 0.6 MPa was selected as the optimal reaction condition for oxygen delignification.

In summary, the optimal conditions for the oxygen delignification of Super-Arundo donax kraft pulp are as follows: alkali dosages of 3%, bleaching temperature of 100 °C, reaction time of 60 min, oxygen pressure of 0.6 MPa, and magnesium sulfate dosage of 0.6%.

### 3.2. Modeling of H_2_O_2_-Enhanced Oxygen Delignification

#### 3.2.1. Results and Model Equations of H_2_O_2_-Enhanced Oxygen Delignification

Table 1 presents the results of 17 sets of H_2_O_2_-enhanced oxygen delignification experiments designed by Design-Expert software.

A model uses data from numerous experiments combined with corresponding mathematical formulas to express and predict the relationship between the expected value(s) of one or more outcomes and one or more controllable independent variables. The following results were achieved for the response surface models characterizing pulp yield and brightness, with alkali dosage, temperature, and H_2_O_2_ dosage as the influencing factors:

(3) $$PY\;=\;90.50-3.84A-1.53T-1.34H-3.00A^{2}-0.2665T^{2}-1.00H^{2}\;+\;0.5600AT\;+\;0.7725AH\;+\;0.2225TH$$

(4) $$PY=89.95850+9.79525A-0.049562T-0.375750H-2.99650A^{2}-0.000666T^{2}\;-0.251000H^{2}+0.028000AT+0.386250AH+0.005563TH$$

(5) $$BR=53.03+1.22A+1.36T+2.29H-0.4687A^{2}-0.5796T^{2}-1.13H^{2}+0.8217AT-0.1584AH+0.2942TH$$

(6) $$BR=29.05500+0.240920A+0.205126T+2.91854H-0.468732A^{2}-0.001449T^{2}-0.283633H^{2}+0.041084AT\;-0.079212AH+0.007354TH\;$$

Among them, the constant term represents the baseline value of the dependent variable; the linear terms denote the linear main effects of independent variables on the dependent variable—for example, when other variables are fixed, each 1-unit increase in A leads to an average change of 9.79525 in pulp yield; the quadratic terms describe the nonlinear main effects of a single independent variable on the dependent variable—if the corresponding coefficient is >0, the independent and dependent variables exhibit a parabola relationship opening upward (decreasing first and then increasing), and if the coefficient is <0, it is a parabola relationship opening downward (increasing first and then decreasing); the two-way interaction terms illustrate the synergistic effects of two independent variables on y, where the influence of one variable on the dependent variable changes with the variation of the other variable.

#### 3.2.2. Model Analysis and Validation of H_2_O_2_-Enhanced Oxygen Delignification

Table 2 shows the analysis of variance (ANOVA) and core fitting statistics. These data correspond to the fitted models of pulp yield and brightness during H_2_O_2_-enhanced oxygen delignification of Super-Arundo donax kraft pulp. Cor Total SS refers to the corrected total Sum of Squares, reflecting the overall variation range of the response value y. Model SS (Model Sum of Squares) represents the variation range of the response value caused by changes in process parameters. The closer the Model SS and Cor Total SS of a response value are, the more significant the influence of the experimental factors under investigation on the experimental results. For pulp yield, the Model SS is 199.31 and the Cor Total SS is 200.48; for brightness, the Model SS is 80.46 and the Cor Total SS is 81.87. Meanwhile, a small Lack of Fit SS (Lack of Fit Sum of Squares) indicates no obvious fitting defects. The Model F-values for pulp yield and brightness are 133.34 and 44.17, respectively, and the Model *p*-values for both are <0.001, indicating that a good functional relationship can be established for the dependent variables with the independent variables. Additionally, the small F-value of Lack of Fit implies low systematic error, reflecting that no other key factors are omitted in model establishment. A *p*-value > 0.05 for Lack of Fit indicates it is not significant, and no model modification is required [35]. In general, the regression models established for H_2_O_2_-enhanced oxygen delignification can accurately reflect the functional relationships between the three factors (alkali dosage, temperature, and H_2_O_2_ dosage) and pulp yield/brightness, with good model fitting.

Roughly speaking, R^2^ (coefficient of determination), AR^2^ (Adjusted R^2^), and PR^2^ (Predicted R^2^) in the model respectively indicate the fitting degree of existing data, the quality of model fitting, and the accuracy of predicting new data. An ideal model should satisfy values close to 1, a difference between R^2^ and AR^2^ less than 0.05, and a difference between AR^2^ and PR^2^ less than 0.2. For pulp yield in this experiment, R^2^ is 0.9942, AR^2^ is 0.9867, and PR^2^ reaches 0.9260. For pulp brightness, R^2^, AR^2^, and PR^2^ are 0.9827, 0.9605, and 0.7965, respectively, all meeting the above conditions. Although a higher R^2^ (closer to 1) indicates better fitting of the model to existing experimental data, it has limitations: R^2^ will increase as long as additional factor terms are added to the model, failing to reflect the actual value of the model. Therefore, residual verification is required to confirm the rationality of the model.

**Figure 5:**
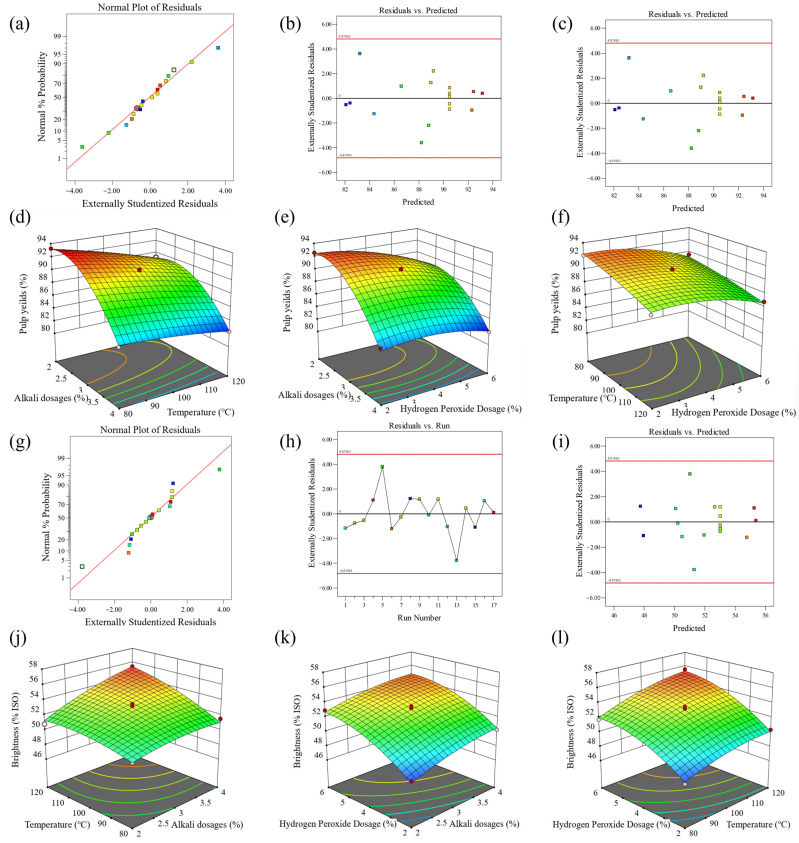
Diagnostics and model graphs for pulp yield: (**a**) normal plot of residuals, (**b**) residuals versus predicted, (**c**) residuals versus run numbers, (**d**–**f**) response surfaces and contour plots. And diagnostics and model graphs for brightness: (**g**) normal plot of residuals, (**h**) residuals versus predicted, (**i**) residuals versus run numbers, (**j**–**l**) response surfaces and contour plots.

For the pulp yield and brightness models, the normal probability plots of residuals, the association of residuals with predicted responses, and the correlation of residuals with experimental runs are presented in Figure 5a–c,g–i. As shown in Figure 5a,g, the residuals and error terms in the two normal probability plots are approximately distributed in a straight line. The red solid line represents the theoretical normal distribution reference line, against which the normality of the externally studentized residuals is assessed. Distinct colored symbols (squares, circles) are used to distinguish individual data points, each corresponding to the externally studentized residual of a single observation. The consistent clustering of points around the red line confirms that the residuals approximately follow a normal distribution; Figure 5b,c,h,i show that the residual points are randomly distributed around the scale line without an obvious trend, indicating stable fitting.

Four sets of random experiments (Table 3) were conducted to verify the pulp yield and brightness calculated by the formulas. Comparison with the actual pulp yield and brightness obtained from experiments showed that the differences between them were very small, not exceeding 0.2%. Therefore, the predicted results of the obtained equations are reasonable.

#### 3.2.3. Parameter Analysis in the Model Equations of H_2_O_2_-Enhanced Oxygen Delignification

It can be seen from the final equations based on coded factors that positive and negative signs indicate the synergistic and antagonistic effects between independent variables and the expected value, respectively [36]. Increases in alkali dosage, temperature, or H_2_O_2_ dosage all exhibit an antagonistic effect on pulp yield, which is reflected in the decrease in yield in the experimental data. The magnitude of the coefficients indicates the degree of influence: 3.84 > 1.53 > 1.34. This shows that among the three variables studied, alkali dosage has the greatest impact on pulp yield, while the impacts of temperature and H_2_O_2_ dosage are similar. The increase in alkali dosage enhances the chemical concentration and activates lignin, improving lignin removal efficiency. Meanwhile, the free radicals generated during the process also cause carbohydrate degradation, leading to a greater degree of yield reduction.

The benzene rings in the structural units of lignin are colorless. However, under high-temperature and strong alkaline conditions, the benzene rings in lignin can form various colored quinoid structures that act as chromophores, resulting in a dark color and low brightness of the pulp. For example, p-benzoquinone (1,4-benzoquinone) is yellow, and o-benzoquinone (1,2-benzoquinone) is red. During H_2_O_2_-enhanced oxygen delignification, these quinoid structures are destroyed, converting lignin back into colorless structures. Since O_2_ and H_2_O_2_ share similar reaction conditions, both decompose in an alkaline environment to generate hydroperoxyl anions (HOO^−^), which can react with carbonyl groups and double bonds on the lignin side chains. This not only reduces or eliminates chromophores in lignin but also degrades lignin into small-molecule fragments that can be dissolved, thereby significantly improving the bleaching effect. Thus, adding a small amount of hydrogen peroxide can achieve a whiteness gain of 11.81% ISO. A reaction time of 60 min ensures that the bleaching reaction proceeds sufficiently while remaining industrially feasible, achieving an optimal balance between time and efficiency. However, H_2_O_2_ can also decompose to generate hydroxyl radicals and hydroperoxyl radicals. Under mild conditions, their reaction with carbohydrates is minimal. Under high-temperature conditions, more radicals are produced, leading to severe degradation.

#### 3.2.4. Model Optimization and Validation of H_2_O_2_-Enhanced Oxygen Delignification

The purpose and significance of constructing a model lie in using known experimental data to establish a response model and derive corresponding formulas, thereby enabling software-based model data optimization to obtain the desired response values. The ideal goal of process optimization is to achieve the optimal results with the lowest energy consumption. However, there must be conditional constraints in actual production. For instance, maintaining the temperature in the range of 80~120 °C is critical to achieve a satisfactory reaction rate while mitigating carbohydrate degradation.

Through the optimization of the model formulas, the ideal conditions were obtained as follows: alkali dosage of 2.84%, bleaching temperature of 105 °C, and H_2_O_2_ dosage of 4.85%. Under these conditions, the theoretical pulp yield was 89.82% and the brightness could reach 53.92% ISO. Based on the conditions provided above, the optimized parameters for the H_2_O_2_-enhanced oxygen delignification of Super-Arundo donax kraft pulp are: pulp consistency of 10%, alkali dosage of 2.84%, bleaching temperature of 105 °C, H_2_O_2_ dosage of 4.85%, bleaching time of 60 min, and MgSO_4_ dosage of 0.6%. The final pulp yield was 89.76% and the brightness was 53.85% ISO, which are very close to the predicted results.

### 3.3. SEM, FTIR and XRD Analysis

#### 3.3.1. SEM Photographs

**Figure 6:**
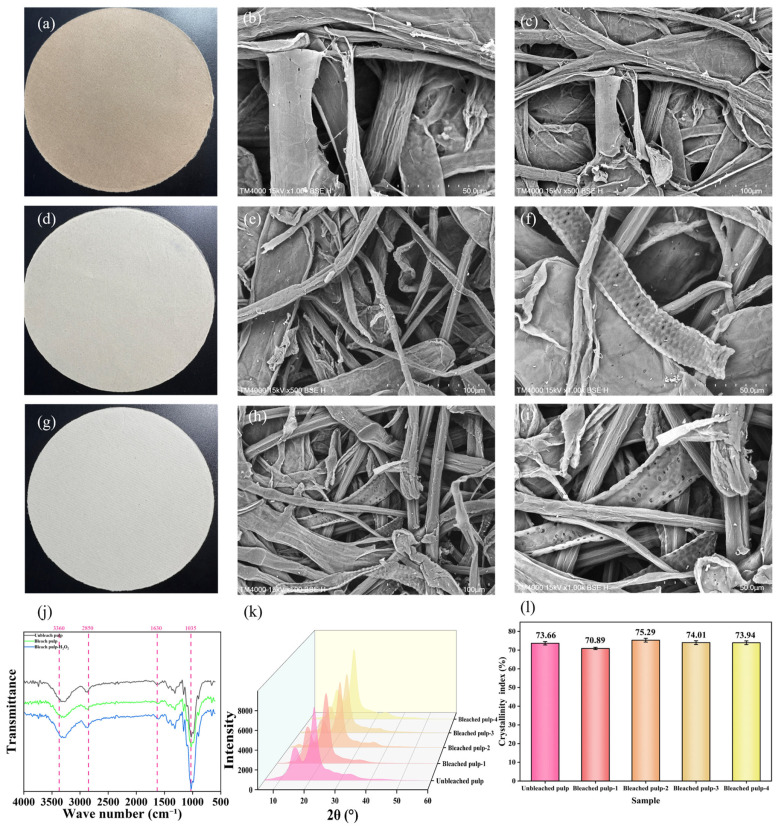
Papers under different conditions. (**a**) Unbleached paper. (**d**) Bleached paper. (**g**) Bleached paper with H_2_O_2_. (**b**,**c**,**e**,**f**,**h**,**i**) SEM images. (**j**) FTIR of three kinds of pulp. (**k**) The XRD patterns of various pulps processed via Maud. (**l**) Crystallinity index of different pulps.

Figure 6a,d,g shows the handsheets made from unbleached pulp, oxygen-delignified pulp, and H_2_O_2_-enhanced oxygen-delignified pulp, respectively. The right of each handsheet shows the corresponding scanning electron microscope (SEM) images. As illustrated in Figure 6, with the progression of the bleaching process, more and more holes appear on the pulp fibers, becoming increasingly dense. This indicates that during the bleaching process, lignin is dissolved and degraded from the fiber matrix, and the remaining holes are impregnated by the chemical solution. Consequently, the bleaching effect is enhanced and the brightness is improved.

#### 3.3.2. FTIR Spectra

Figure 6j shows the FTIR spectra of three pulp samples: unbleached, oxygen-delignified, and H_2_O_2_-enhanced oxygen-delignified. Pulps bleached by different methods exhibited varying degrees of changes in their FTIR spectra. For the absorption peak at 1035 cm^−1^, the blue curve (H_2_O_2_-bleached pulp) showed a sharp peak shape with high intensity, while the peaks of the red curve (oxygen-delignified pulp) and black curve (unbleached pulp) were less obvious. This indicates that H_2_O_2_ bleaching enhanced the signal of carbohydrate-related peaks, possibly due to more thorough lignin removal, which made the characteristics of carbohydrate structures more prominent. In this region (1150~1200 nm), the spectral signal is associated with the stretching of C-H bonds and the presence of lignin. The unbleached pulp had a distinct absorption peak at 1630 cm^−1^, reflecting the presence of aromatic lignin structures [37]. The intensity of these peaks decreased in bleached pulps, with the blue curve showing the weakest peak, confirming that H_2_O_2_ bleaching had a more significant degradation effect on aromatic lignin. The unbleached pulp exhibited a more prominent peak at 2850 cm^−1^, indicating a higher content of aliphatic lignin or extractives. The decreased peak intensity in bleached pulps demonstrated that the bleaching process effectively removed aliphatic components. The peak at 3360 cm^−1^ is attributed to the O–H stretching vibration of hydroxyl (-OH) groups in cellulose, hemicellulose, and lignin. No significant change was observed at this peak position. Although lignin was removed during the bleaching process, hydroxyl groups in the system were mainly derived from cellulose and hemicellulose. The change in hydroxyl groups caused by lignin removal was relatively small, so no significant variation was observed in the hydroxyl absorption peak at 3360 cm^−1^. By comparing the three curves: the unbleached pulp shows a broader and stronger absorption peak, indicating a higher hydroxyl content in the unbleached pulp (possibly because the hydroxyl groups in lignin and carbohydrates have not been largely removed); the absorption peaks of the bleached pulp and H_2_O_2_-enhanced bleached pulp are significantly weakened, especially the H_2_O_2_-enhanced group, indicating that during the bleaching process, lignin is removed and hydroxyl groups are oxidized or reacted, resulting in a decrease in hydroxyl content.

#### 3.3.3. XRD Analysis

X-ray diffraction (XRD) measurements were undertaken on both Super-Arundo donax kraft pulp as well as its bleached pulp. The bleached pulp-1 was not added with H_2_O_2_, the bleached pulp-2 (2% alkali dosage and 2% H_2_O_2_), the bleached pulp-3 (3% alkali dosage and 4% H_2_O_2_), the bleached pulp-4 (4% alkali dosage and 6% H_2_O_2_), all experimental groups were subjected to a temperature of 100 °C. The results are shown in Figure 6k,l.

All pulps exhibited three characteristic peaks around 2θ = 15.9°, 22.6°, and 34.5°, corresponding to the structure of natural cellulose (Cellulose I). This indicates that oxygen delignification and H_2_O_2_-enhanced oxygen delignification did not alter the main crystalline structure of cellulose. In the course of bleaching, the oxidant inevitably attacks the amorphous regions of cellulose and damages the edge structures of the crystalline regions, leading to a decrease in the crystallinity and DP of the fibers. After adding H_2_O_2_, more lignin can be removed, resulting in a slight upward trend in the overall crystallinity of cellulose. However, excessively strong oxidation conditions will still act on the fibers, causing an overall downward trend in crystallinity. This also indicates that the addition of hydrogen peroxide is beneficial to lignin removal.

### 3.4. Comparative Analysis of Different Raw Materials

In this study, the oxygen-alkali bleaching of Super-Arundo donax kraft pulp was compared with those of bleaching processes for several other papermaking raw materials, which further illustrates the advantages and development prospects of Super-Arundo donax as a non-wood raw material in the pulp and paper industry. The bleaching processes for all raw materials are as follows: alkali dosage of 3%, temperature of 100 °C, oxygen pressure of 0.6 MPa, and reaction time of 60 min. The results are shown in Table 4.

Although the brightness of Super-Arundo donax kraft pulp is slightly lower than that of woody raw materials after treatment under the same bleaching conditions, the mechanical strength of the resulting paper is comparable. The tensile strength index, bursting index and tear index of bleached Super-Arundo donax kraft pulp were 60.92 N·m/g, 4.16 kPa·m^2^/g, and 5.45 mN·m^2^/g respectively. The tensile strength index, bursting index and tear index of bleached eucalyptus kraft pulp were 55.6 N·m/g, 4.64 kPa·m^2^/g, and 7.86 mN·m^2^/g respectively. Compared with other non-wood raw materials, the mechanical strength of both bleached reed kraft pulp and bleached sasa kraft pulp is far inferior to that of bleached Super-Arundo donax kraft pulp. Wheat straw pulp exhibits lower brightness and viscosity than super golden reed kraft pulp. Moreover, wheat straw pulp suffers from severe silica interference in black liquor, which hinders alkali recovery and raises production costs. Unbleached bagasse pulp has a relatively high initial brightness and thus achieves higher bleached brightness. Nevertheless, it is characterized by low yield, low viscosity, and insufficient pulp strength, making it difficult to meet the performance requirements of high-grade paper products. The advantages of Super-Arundo donax are reflected in three dimensions: raw material characteristics, production efficiency, and ecological value. The features slender fibers with high cellulose content, and its fiber morphology is close to that of hardwood. The resultant paper pulp demonstrates excellent performance in key indicators. Super-Arundo donax has a short growth cycle, which is tolerant to barren soil and salinization, thus suitable for cultivation on marginal lands. It boasts large biomass and low planting and management costs, which can ensure stable raw material supply, alleviate the shortage of wood resources, and reduce the raw material procurement costs of papermaking enterprises. No large amounts of chemical fertilizers or pesticides are required during cultivation. Moreover, it can stabilize soil, conserve water, and absorb carbon dioxide. As a non-wood raw material, it helps reduce the logging of natural forests, conforming to the green and low-carbon development trend of the papermaking industry. This indicates that Super-Arundo donax is a viable alternative to other non-wood raw materials for pulp and paper production.

## 4. Conclusions

This study explored the feasibility of Super-Arundo donax for pulp production. It is a novel non-wood plant fiber and a sustainable alternative to traditional wood-based raw materials. Initial single-factor experiments were conducted to evaluate the effects of key bleaching parameters, including alkali dosage, reaction time, temperature, and oxygen pressure. Based on comprehensive assessment of pulp yield, brightness, Kappa number, viscosity, and handsheet mechanical properties, the optimal conditions for conventional oxygen delignification were determined as follows: alkali dosage of 3.0%, bleaching time of 60 min, temperature of 100 °C, oxygen pressure of 0.6 MPa, and MgSO_4_ dosage of 0.6%. Under these conditions, the bleached pulp achieved a high yield of 91.58%, brightness of 42.04% ISO, Kappa number of 7.58, viscosity of 1094.5 mL/g, tensile index of 60.92 N·m/g, bursting index of 4.16 kPa·m^2^/g, and tear index of 5.45 mN·m^2^/g. To further enhance delignification efficiency and brightness, an H_2_O_2_-enhanced oxygen delignification process was subsequently explored. The optimized conditions for the H_2_O_2_-enhanced process were identified as: pulp consistency of 10%, alkali dosage of 2.84%, H_2_O_2_ dosage of 4.85%, temperature of 105 °C, bleaching time of 60 min, and MgSO_4_ dosage of 0.6%. Under these refined conditions, the pulp yield was 89.76%, with a significantly improved brightness of 53.85% ISO. Collectively, Super-Arundo donax is a promising raw material for chemical pulping. Meanwhile, H_2_O_2_-enhanced oxygen delignification can effectively improve pulp brightness while maintaining acceptable yield. This provides a viable pathway toward sustainable non-wood fiber utilization in the pulp and paper industry.

## Figures and Tables

**Table 1:** Central composite design matrix.

Trial	Alkali Dosages (%)	Temperature (°C)	H_2_O_2_ Dosage (%)	Pulp Yields (%)	Brightness (%ISO)
1	2	80	4	93.25	50.20
2	4	80	4	84.12	51.52
3	2	120	4	89.23	50.80
4	4	120	4	82.34	55.41
5	2	100	2	92.56	48.02
6	4	100	2	83.67	50.26
7	2	100	6	87.78	52.92
8	4	100	6	81.98	54.52
9	3	80	2	92.12	47.72
10	3	120	2	88.45	50.32
11	3	80	6	89.56	51.73
12	3	120	6	86.78	55.51
13	3	100	4	90.65	53.23
14	3	100	4	90.32	52.72
15	3	100	4	90.81	52.80
16	3	100	4	90.54	52.92
17	3	100	4	90.17	53.50

**Table 2:** ANOVA and R^2^ statistics for the fitted oxygen delignification models.

Source	Sum of Squares	DF	Mean Square	F-Value	*p*-Value	R^2^	Adjusted R^2^	Predicted R^2^
Pulp Yield	-	-	-	-	-	-	-	-
Model	199.31	9	22.15	133.34	<0.001	0.9942	0.9867	0.9260
Residual	1.16	7	0.1661	-	-	-	-	-
Lack of Fit	0.9011	3	0.3004	4.59	0.0874	-	-	-
Pure error	0.2615	4	0.0654	-	-	-	-	-
Cor Total	200.48	16	-	-	-	-	-	-
Brightness	-	-	-	-	-	-	-	-
Model	80.46	9	8.94	44.17	<0.001	-	-	-
Residual	1.42	7	0.2024	-	-	-	-	-
Lack of Fit	1.00	3	0.3335	3.21	0.1449	0.9827	0.9605	0.7965
Pure error	0.4160	4	0.1040	-	-	-	-	-
Cor Total	81.87	16	-	-	-	-	-	-

**Table 3:** Model validation.

Trial	Alkali Dosages (%)	Temperature (°C)	H_2_O_2_ Dosages (%)	Theoretical Pulp Yields (%)	Actual Pulp Yields (%)	Theoretical Brightness (%ISO)	Actual Brightness (%ISO)
1	2	80	2	94.50	94.35	46.93	46.92
2	4	120	6	81.08	81.15	56.68	56.38
3	3	80	5	90.73	90.81	51.81	51.74
4	2.5	100	4	91.67	91.60	52.31	52.24

**Table 4:** Bleaching performance of some papermaking raw materials.

	Brightness (% ISO)	Viscosity (mL/g)	Kappa Number	Tensile Index (N·m/g)	Bursting Index (kPa·m^2^/g)	Tear Index (mN·m^2^/g)
Super-Arundo donax kraft pulp	42.04	1094.5	7.58	60.92	4.16	5.45
Reed kraft pulp	47.81	943.0	6.60	27.68	1.44	-
Eucalyptus kraft pulp	58.10	793.0	6.77	55.60	4.64	7.86
Poplar kraft pulp	63.40	833.0	4.80	28.50	-	2.48
Sasa kraft pulp	40.50	963.0	14.30	-	-	-
Wheat straw soda-AQ pulp	40.64	989.0	6.95	-	-	-
Bagasse soda-AQ pulp	48.30	790.0	6.82	-	-	-

## Data Availability

The original contributions presented in this study are included in the article/Supplementary Material. Further inquiries can be directed to the corresponding authors.

## Supplementary Materials

The following supporting information can be downloaded at: https://www.mdpi.com/article/10.3390/polym18060750/s1, Table S1: Design of central composite experiment; Table S2: The effect of alkali dosages on oxygen delignification; Table S3: The effect of temperature on oxygen delignification; Table S4: The effect of time on oxygen delignification; Table S5: The effect of pressure of oxygen on oxygen delignification.
