# Supplementary material for: Optimization of Bleaching Process and Evaluation of Pulp Performance for Super-Arundo Donax Kraft Pulp
Source: Polymers (Basel). 2026 Mar 19;18(6):750. doi: 10.3390/polym18060750 (PMC13030791; doi:10.3390/polym18060750)
Supplement: Supplementary file 1 [file polymers-18-00750-s001.zip › polymers-4201170-supplementary.pdf]

Supplementary materials

Optimization of bleaching process and evaluation of pulp performance for Super-Arundo donax kraft pulp

Zhangming Cai [1], Xingxiang Ji [1], Jie Liang [1], Zhongjian Tian [1,*], Jingpeng Zhou [2,*]

[1] *State Key Laboratory of Green Papermaking and Resource Recycling, Qilu University of Technology (Shandong Academy of Sciences), Jinan 250353, China*

[2] *Shandong Huatai Paper Co., Ltd. Dongying 257335, China*

* Corresponding author. E-mail address: tianzhj@qlu.edu.cn (Z.T.); zjpzll@163.com (J.Z.)

Supplementary Table S1. Design of central composite experiment.

Trial	Alkali dosages (%)	Temperature (℃)	H2O2 dosages (%)
1	2	80	4
2	4	80	4
3	2	120	4
4	4	120	4
5	2	100	2
6	4	100	2
7	2	100	6
8	4	100	6
9	3	80	2
10	3	120	2
11	3	80	6
12	3	120	6
13	3	100	4
14	3	100	4
15	3	100	4
16	3	100	4
17	3	100	4

Supplementary Table S2. The effect of alkali dosages on oxygen delignification.

Alkali dosages (%)	2	2.5	3	3.5	4
Pulp yields (%)	93.55	92.73	91.58	89.07	86.34
Brightness (%ISO)	34.7	36.34	42.04	42.6	42.89
Kappa number	8.03	7.88	7.58	6.86	6.36
Viscosity (mL/g)	1161	1124	1094.5	1079.5	1069.5
Klason lignin (%)	0.69	0.60	0.51	0.45	0.39
Acid-soluble Lignin (%)	0.59	0.56	0.51	0.48	0.43
Tensile index (N • m/g)	64.19	62.02	60.92	59.18	58.60
Bursting index (kPa • m^2/g)	4.30	4.26	4.16	4.03	3.84
Tear index (mN • m^2/g)	6.43	5.71	5.45	5.24	5.10
Arabinose (g/L)	0.0475	0.0479	0.0495	0.0519	0.0600
Glucose (g/L)	1.2884	1.2943	1.2962	1.3251	1.3388
Xylose (g/L)	0.9137	0.9433	0.9857	1.0011	1.0164

Supplementary Table S3. The effect of temperature on oxygen delignification.

Temperature (°C)	80	90	100	110	120
Pulp yields (%)	93.76	92.67	91.58	90.5	89.48
Brightness (%ISO)	38.76	40.89	42.04	42.15	42.24
Kappa number	8.94	8.18	7.58	6.66	6.06
Viscosity (mL/g)	1163	1143	1094.5	1048	1013.5
Klason lignin (%)	0.67	0.57	0.51	0.47	0.41
Acid-soluble Lignin (%)	0.66	0.61	0.51	0.46	0.36
Tensile index (N·m/g)	63.42	61.91	60.92	59.83	58.76
Bursting index (kPa·m^2/g)	4.35	4.23	4.16	3.90	3.73
Tear index (mN·m^2/g)	5.77	5.56	5.45	5.33	5.26
Arabinose (g/L)	0.0412	0.0428	0.0495	0.0391	0.0362
Glucose (g/L)	1.0372	1.0227	1.2962	0.9543	0.8752
Xylose (g/L)	0.8235	0.8253	0.9857	0.6802	0.6485

Supplementary Table S4. The effect of time on oxygen delignification.

Time (min)	20	40	60	80	100
Pulp yields (%)	93.2	92.04	91.58	90.88	90.58
Brightness (% ISO)	38.47	40.55	42.04	42.25	42.36
Kappa number	8.79	8.27	7.58	7.23	6.87
Viscosity (mL/g)	1111.5	1104.5	1094.5	1073.5	1027
Klason lignin (%)	0.74	0.59	0.51	0.45	0.40
Acid-soluble Lignin (%)	0.66	0.61	0.51	0.46	0.39
Tensile index (N·m/g)	63.76	62.20	60.92	59.86	58.37
Bursting index (kPa·m^2/g)	4.77	4.53	4.16	4.07	3.98
Tear index (mN·m^2/g)	6.03	5.87	5.45	5.37	5.27
Arabinose (g/L)	0.0452	0.0486	0.0495	0.0501	0.0503
Glucose (g/L)	1.2304	1.2763	1.2962	1.3188	1.3193
Xylose (g/L)	0.9281	0.9786	0.9857	0.988	1.0255

Supplementary Table S5. The effect of pressure of oxygen on oxygen delignification.

Pressure of oxygen (MPa)	0.4	0.5	0.6	0.7	0.8
Pulp yields (%)	92.64	92.27	91.58	90.85	89.82
Brightness (% ISO)	41.67	41.83	42.04	42.21	42.26
Kappa number	9.24	8.48	7.58	7.27	6.67
Viscosity (mL/g)	1118.5	1107	1094.5	1079.5	1056
Klason lignin (%)	0.62	0.56	0.51	0.46	0.43
Acid-soluble Lignin (%)	0.52	0.52	0.51	0.50	0.50
Tensile index (N·m/g)	63.47	62.41	60.92	60.65	60.56
Bursting index (kPa·m^2/g)	4.25	4.25	4.16	4.14	4.11
Tear index (mN·m^2/g)	5.67	5.52	5.45	5.33	5.21
Arabinose (g/L)	0.0485	0.0489	0.0495	0.0540	0.0577
Glucose (g/L)	1.1846	1.2248	1.2962	1.3359	1.339
Xylose (g/L)	0.9615	0.9731	0.9857	0.9927	0.9962
